# Reduced MCMV Δm157 viral clearance in the absence of TSAd

**DOI:** 10.1038/srep09219

**Published:** 2015-03-18

**Authors:** P. Moussa, G. Abrahamsen, N. Fodil, R. P. Gopalakrishnan, M. Mancini, E. Dissen, P. C. Sæther, S. A. Wiltshire, G. A. Boivin, G. Caignard, A. Spurkland, S. M. Vidal

**Affiliations:** 1 Department of Human Genetics and Department of Microbiology and Immunology, McGill University, Life Sciences Complex Montreal, QC, Canada; 2 Department of Anatomy, Institute of Basic Medical Sciences, University of Oslo, Oslo, Norway

## Abstract

The T cell specific adapter protein (TSAd) is expressed in activated T cells and NK cells. While TSAd is beginning to emerge as a critical regulator of Lck and Itk activity in T cells, its role in NK cells has not yet been explored. Here we have examined susceptibility to virus infections in a murine model using various viral infection models. We report that TSAd-deficient mice display reduced clearance of murine cytomegalovirus (MCMV) that lack the viral MHC class I homologue m157, which is critical for Ly49H-mediated NK cell recognition of infected cells. In this infection model, NK cells contribute in the early stages of the disease, whereas CD8+ T cells are critical for viral clearance. We found that mice infected with MCMV Δm157 displayed reduced viral clearance in the spleen as well as reduced proliferation in spleen NK cells and CD8+ T cells in the absence of TSAd. Though no other immunophenotype was detected in the infection models tested, these data suggests that in the absence of the Ly49H ligand activation, NK cell and CD8+ T cell responses may be compromised in TSAd-deficient mice.

The recognition of antigens by the T cell receptor (TCR) is crucial in order to mediate T cell dependent responses. Triggering of the TCR leads to downstream signal propagation through a sequence of phosphorylation events modulated by various adaptor proteins. One of these adaptors, encoded by the *Sh2d2a* gene, is T cell specific adapter protein (TSAd), which is upregulated in activated T cells^1^.

T cells from *Sh2d2a* *^−/−^* mice display impaired production of IFNγ upon TCR stimulation *in vitro*^2^ and in *vivo*^3^. Early characterization of *Sh2d2a* *^−/−^* mice indicated that TSAd regulated autoimmunity, as T cells from *Sh2d2a* *^−/−^* mice were more resistant to antigen-induced cell death in vivo and older *Sh2d2a* *^−/−^* mice developed lupus-like symptoms^3^. Recently, improved T cell mediated protection against a transplanted myeloma in *Sh2d2a^−/−^* mice has been observed indicating that TSAd has a modulatory role in T cell mediated immune surveillance of cancer^4^. Though the characterisation of TSAd's function *in vivo* remains incomplete, these studies of TSAd suggest that the adaptor may be an important regulator of normal T cell function.

Biochemical characterization of TSAd's role in T cell activation has revealed that TSAd modulates the activity of Lck through multi-site docking of Lck to TSAd^5^. TSAd interacts with Itk, and primes Itk for phosphorylation by Lck^6^. It is thus likely that TSAd modulates signalling in activated T cells via interaction with Lck and Itk^5^,^6^,^7^,^8^.

Although TSAd is also highly expressed in NK cells^9^, the role of TSAd in NK cell function has not been explored. TSAd expression is induced upon activation of both T and NK cells and many of the known interaction partners for TSAd such as Lck, Itk, Src and MEKK2^2^,^10^,^11^,^12^,^13^ are also expressed in NK cells. Importantly, Lck and Itk are expressed almost solely in T and NK cells and are important for efficient NK cell cytotoxicity and survival^12^,^13^,^14^,^15^,^16^.

There is compelling evidence for the role of NK cells against virus infections such as coxsackievirus B3^17^, influenza virus^18^ as well as a potential role in herpes simplex virus 1 (HSV1) infection^19^. The best characterized model however remains the functional impact of NK cells against mouse cytomegalovirus (MCMV). In C57BL/6 mice, NK cells expressing the activating receptor Ly49H are responsible for the control of MCMV replication^20^,^21^. Ly49H binds to the viral glycoprotein m157 which is expressed at the surface of infected cells within hours of infection^22^. This binding triggers signaling pathways that activate NK cell effector functions such as IFNγ secretion, release of cytotoxic granules containing perforin and granzymeB, as wells as proliferation of Ly49H+ NK cell subsets^22^. The antiviral response to MCMV also involves CD4+ T cells, NK-T cells as well as neutrophils. CD4+ T cells are critical for controling virus load in the salivary glands, but not the spleen, lung or liver^23^,^24^. A recent report demonstrated that depletion of neutrophils increases virus load in several organs including liver, salivary gland and lungs but not in the spleen^25^. NK-T cells are activated by MCMV and have been shown to synergize with NK cells for the clearance of MCMV in a BALB/c model, although this occurs principally in the liver^26^.

Finally, an MCMV strain that lacks the m157 protein (Δm157 MCMV) and thus bypasses the stringent Ly49H NK cell response^27^ has enabled the discovery of CD8+ T cell and NK cell cross talk during viral infection^28^. While CD8+ T cells have only a minor role in the control of wild type MCMV, they have a critical function in the clearance of m157-deficient virus^28^. This detailed understanding of MCMV and its interaction with NK and CD8+ T cells together with availability of *Sh2d2a* *^−/−^* mice provide ideal tools for investigating the function of TSAd in the context of infection.

Here, we report the use of various virus infection models, including *Δm157* MCMV, to assess the function of TSAd during a primary viral infection. Absence of an altered anti-virus response in wild type MCMV infected *Sh2d2a* *^−/−^* mice, and impaired viral clearance in *Δm157* MCMV infected *Sh2d2a* *^−/−^* mice indicates that TSAd does not modulate NK cell function through the Ly49H activating pathway and suggests that TSAd is implicated in T cell mediated elimination of virus infected cells.

## Results and discussion

### TSAd is expressed in NK cells

**Figure 1:**
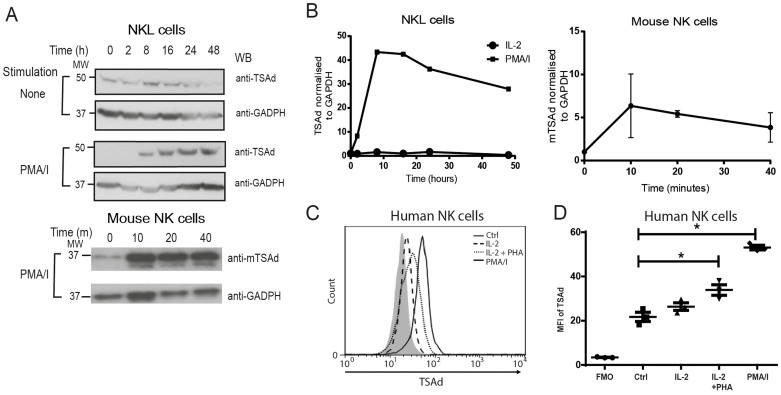
TSAd expression in NK cells. (A) Human NK-L cell line was stimulated with 200 U/ml of IL-2 or 100 ng/ml of PMA and 500 ng/ml of Ionomycin (PMA/I) for the indicated time points. The cells were then lysed and the expression of TSAd was analysed by Western blot. Glyceraldehyde-3-phosphate dehydrogenase (GAPDH) served as loading control for total protein level (Top). Mouse NK cells prepared from total splenocytes and cultured with recombinant human IL-2 (1000 U/mL) for 6 to 8 days were stimulated with PMA/I for the indicated time-points and TSAd expression was monitored by Western blot as indicated above (Bottom). (B) Densitometry analysis was performed on the Western blot data, shown as TSAd/GAPDH ratio. (C and D) PBMC from healthy volunteers were stimulated with 200 U/ml of IL-2 or 200 μg/ml of PHA along with 200 U/ml of IL-2 or 100 ng/ml of PMA and 500 ng/ml of Ionomycin for 24 h. (C) TSAd expression in NK cells (CD3-, CD56+) from PBMC were analysed by flow cytometry. (D) Graphical representation of the TSAd expression levels in human CD3-CD56+ cells +/− SEM (*p < 0.05). Each dot represents an individual donor.

To confirm expression of TSAd in activated NK cells from C57BL/6 *Sh2d2a* *^+/+^* mice, splenic NK cells were cultured in the presence IL-2 for six to eight days. Western blot analysis demonstrated expression of TSAd in IL-2 activated NK cells, and increased expression following incubation in a cocktail of PMA plus ionomycin. Similarly, the human NK cell line NKL (cultured in IL-2) demonstrated increased expression of TSAd following induction with PMA/ionomycin as analysed by western blot (Figure 1A–B). The mouse NK cells displayed a rapid increase in TSAd expression, evident as early as 10 minutes following PMA/ionomycin stimulation indicating that the expression of TSAd is regulated also at the translational level as reported earlier^29^. In contrast, the human NKL cell line displayed a slower response, with TSAd expression peaking eight hours after stimulation (Figure 1A–B). The amount of TSAd in primary human NK cells from blood as analysed by flow cytometry was not affected by addition of IL-2, However, a significant increase in amount of TSAd was observed upon treatment with IL-2/PHA or PMA/Ionomycin (Figure 1C–D).

### TSAd deficient mice display reduced clearance of Δm157 MCMV infection in the spleen

**Figure 2:**
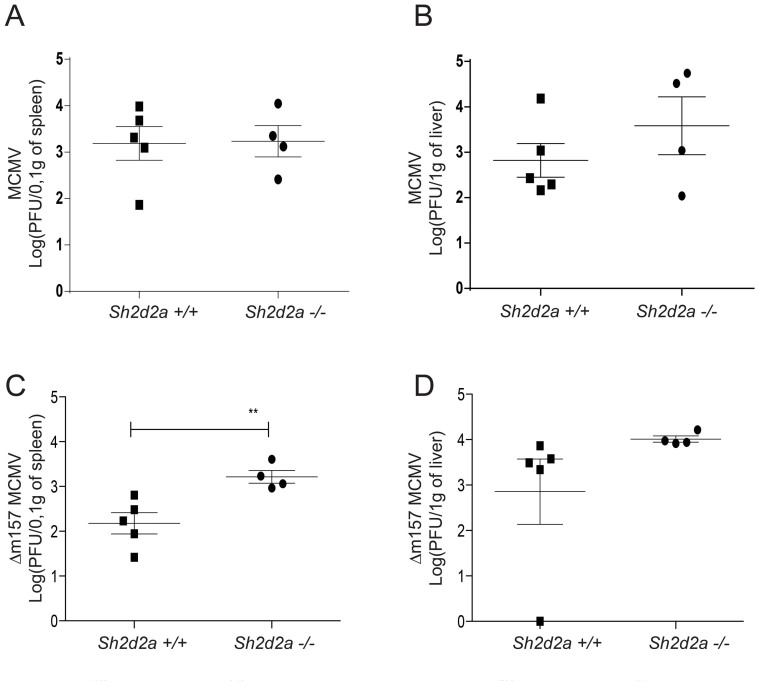
*Sh2d2a* genotype influences host control of MCMV Δm157 but not MCMV virus replication. *Sh2d2a*^+/+^ and *Sh2d2a* *^−/−^* littermates were infected with 7000 PFU of MCMV (Smith strain) or 2 * 10^6^ PFU of MCMV Δm157 virus via the intravenous route. (A–B) At day 4 p.i. mice were sacrificed and the MCMV viral titers from A) spleens and B) livers were determined by standard plaque assays using BALB/c mouse embryonic fibroblasts. (C–D) After MCMV Δm157 infection, viral titers were determined 5 days post-infection in the spleen (C) and liver (D) by standard plaque assays using BALB/c mouse embryonic fibroblasts. Each dot represents an individual mouse and results are expressed as mean +/− SEM. (**p < 0.005). One out of two representative experiments is shown.

In order to determine if TSAd influences host resistance to MCMV infection, *Sh2d2a* *^−/−^* mice and their *Sh2d2a* ^+/+^ littermates were infected with both wild type (Smith strain) MCMV and the *Δm157* MCMV strain, lacking the *m*157 gene, and viral titers in spleen and liver were determined. Infection with MCMV at day 4 did not show a difference between *Sh2d2a^−/−^* and wild type mice neither in the spleen nor the liver (Figure 2A–B). In contrast, 5 days post infection with *Δm157* MCMV, the *Sh2d2a* *^−/−^* mice displayed higher viral titers in the spleen but not the liver, than the *Sh2d2a* ^+/+^ mice (Figure 2C–D). NK cells are critical in clearing MCMV via recognition of the viral MHC homologue m157 by Ly49H^28^,^30^. The observation that wild type MCMV was cleared equally well in TSAd deficient mice and wild type mice (Figure 2A–B), indicates that TSAd is not critical for clearance of the virus by Ly49H-dependent NK cells.

MCMV infection of mice lacking Ly49H (B6. *Ly49h*-deficient mice) leads to high splenic viral titer, disorganization of the spleen architecture and dramatic attrition of lymphocytes during the first three days after infection^30^. In absence of Ly49H signaling, however, CD8+ T cells have been shown to be critical for the control of infection^28^,^30^. This was demonstrated in a model whereby C57BL/6 mice are infected with *Δm157* MCMV^28^,^30^. Already by day 4 post-infection, CD8+ T cells express activation markers and, by day 7, there is robust proliferation and cytokine secretion of antigen-specific CD8+ T cells^28^,^30^.

TSAd is highly expressed in activated T cells. Thus the reduced clearance of *Δm157* MCMV in the spleens of *Sh2d2a* *^−/−^* mice could be caused by deficiency in CD8+ T cell activation or function. However, an early NK cell response modulates CD8+ T cell function during *Δm157* MCMV infection^28^. Deficient NK cell function could also explain the reduced viral clearance in the spleen. Thus, the higher *Δm157* MCMV titers seen in *Sh2d2a* *^−/−^* mice could be due to defective early NK-CD8+ T cell responses or crosstalk. This dysfunction may be masked in wild type MCMV infection by the potent Ly49H-mediated effect resulting in normal resistance to the virus^28^.

**Figure 3:**
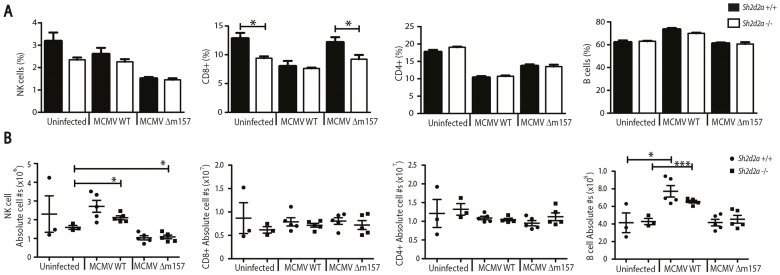
Cell populations of the splenic compartment in *Sh2d2a* ^+/+^ and *Sh2d2a* *^−/−^* mice. Mice were infected with 8000 PFU of WT MCMV or 2 * 10^6^ PFU of Δm157 MCMV. Splenocytes were harvested from uninfected mice or at day 5 post-infection and analyzed for proportions (A) or absolute numbers (B) of spleen cell populations+/− SEM (*p < 0.05; **p < 0.005). Each dot represents an individual mouse One out of two representative experiments is shown.

In order to assess the relative importance of T cells versus NK cells in the reduced *Δm157* MCMV clearance in *Sh2d2a^−/−^* mice, we assessed the absolute numbers and proportions of cell types in the spleen before and after MCMV or *Δm157* MCMV infection in *Sh2d2a* *^−/−^* mice and their wild type littermates (Figure 3). To capture the contribution of both cell types, mice were examined on day 5 post-infection. A minor but significant decrease in the percentage of CD8+ T cells was observed in both non-infected as well as infected *Sh2d2a* *^−/−^* mice compared to their wild type littermates (Figure 3A). Moreover, no significant changes were observed between *Sh2d2a* *^−/−^* and *Sh2d2a* *^+/+^* mice in absolute T cell numbers (Figure 3B). Thus the relative decrease in the percentage of CD8+ T cells is likely due to an influx of another cell type, as there is no difference in the total numbers of CD8+ T cells present in the spleens of *Sh2d2a* ^+/+^ and *Sh2d2a* *^−/−^* mice. Indeed the numbers of CD4+ T cells and B cells are both nominally increased in *Sh2d2a* *^−/−^* mice compared to controls although the difference does not reach significance (Fig. 3B). B cells are required for the initial type I IFN response to MCMV^31^. However, as TSAd is not expressed in B-cells, we find it less likely that these cells are responsible for the reduced clearance of *Δm157* MCMV in *Sh2d2a* *^−/−^* mice. Finally, NK cell numbers show no major differences between the two groups (Figure 3B), suggesting a possible functional defect in NK or CD8+ T cells.

### NK cells from TSAd deficient mice display normal distribution of maturation and activation markers in vivo and in vitro

NK cell maturation was analyzed by the expression of CD27 and CD11b during infection with *Δm157* MCMV in spleen lymphocytes. The order of maturity from least to most mature is CD11b^low^CD27^low^, CD11b^low^CD27^high^, CD11b^high^ CD27^high^ and finally CD11b^high^CD27^low^^32^. As the cell matures along these stages, the NK cell effector functions change, and the levels of CD11b and CD27 expression may thus correlate with how capable the population of NK cells are at clearing MCMV.

**Figure 4:**
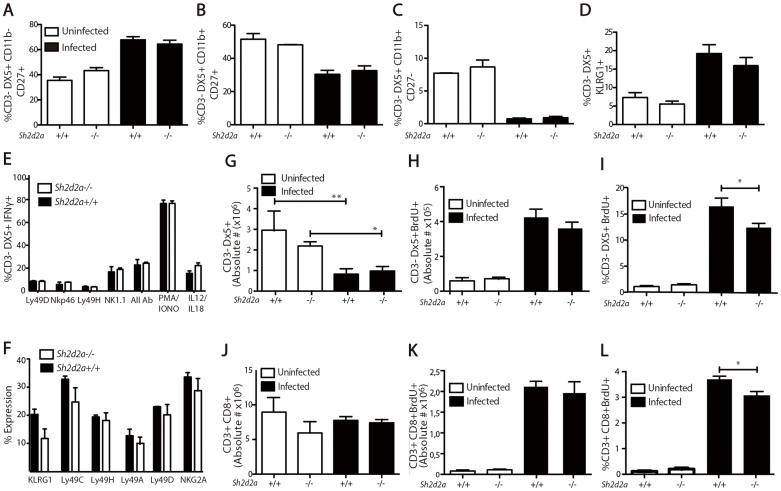
In vivo and in vitro Immunophenotying of NK cells in *Sh2d2a* ^+/+^ and *Sh2d2a* *^−/−^* mice. Total leukocytes were isolated from mice uninfected or infected with 2 * 10^6^ PFU of MCMV Δm157 for 5 days. *Ex vivo* spleen NK cell subsets were gated according to surface expression of maturation markers CD11b and CD27 (A–C) or expression of KLRG1 (D). Explanted lymphocytes from uninfected *Sh2d2a*^+/+^ and *Sh2d2a^−/−^* mice served to determine *in vitro* NK cell expression levels of IFNã upon stimulation with plate bound antibodies (x-axis), PMA/Ionomycin and IL12/IL18 (E). Total number of CD3-DX5+ splenocytes before and after infection with Δm157 for 5 days (F). NK proliferation was monitored by BrdU incorporation and staining with anti-BrdU in CD3-DX5+ splenocytes *ex vivo*. Total number (G) and frequency (H) of BrdU positive cells was determined by flow cytometry. Total number of CD3-CD8+ splenocytes before and after infection with Δm157 for 5 days (I). CD8+ T cell proliferation was monitored by BrdU incorporation and staining with anti-BrdU in CD3-DX5+ splenocytes *ex vivo*. Total number (J) and frequency (K) of BrdU positive cells was determined by flow cytometry. Explanted lymphocytes from uninfected *Sh2d2a*^+/+^ and *Sh2d2a^−/−^* mice served to determine *in vitro* levels of surface activating and inhibitory NK cell receptors (L). Results are expressed as mean +/− SEM. *<0.05, **<0.05.

In order to assess whether there was a difference in NK maturation between *Sh2d2a^−/−^* mice and wild type controls, levels of CD11b and CD27 on the surface of NK cells were analyzed 5 days post infection with *Δm157* MCMV. No differences in any of the stages were observed between *Sh2d2a* *^−/−^* mice and their wild type littermates (Figure 4A–C). The level of the activation marker KLRG1on NK cells also showed no difference prior to and after 5 days of *Δm157* MCMV infection (Figure 4D). Thus, the maturity and activation profile of NK cells are similar in the two groups of mice.

To investigate whether lack of TSAd had an effect on the ability of NK cells to be activated and produce IFNγ, total splenocytes were stimulated *in vitro* with antibodies against the activating NK cell receptors Ly49D, Ly49H, NKp46 or NK1.1. Stimulation with IL12 + IL18 or PMA plus ionomycin served as positive controls. IFNγ expression in NK cells was subsequently measured by flow cytometry. No differences in IFNγ expression were observed between NK cells from *Sh2d2a^−/−^* and wild type donors (Figure 4E). Also, the levels of IFNγ in CD8+ T cells upon IL12 + IL18 or PMA/ionomycin stimulation were not significantly different between *Sh2d2a* *^−/−^* and wild type mice (data not shown).

### Reduced proliferation of TSAd deficient NK and CD8+ T cells during infection with Δm157 MCMV

Although *Sh2d2a* *^−/−^* mice are capable of clearing *MCMV* infection, there may be a defect in the ability of NK cells to proliferate in the absence of TSAd. TSAd's effect on the proliferation of T cells has been disputed. Some studies have found that absence of TSAd reduces T cell proliferation^2^, while others have not^4^,^33^. In order to investigate TSAd's role in NK cell proliferation, BrdU incorporation was measured in splenocytes from *Sh2d2a* *^−/−^* mice and their wild type littermates 5 days after *Δm157* MCMV infection. Five days after infection we observed a significant decrease of 50% on average in the total number of NK cells in infected mice, as it has been previously reported^28^,^30^ (Figure 4F). The number of NK cells during infection was not significantly different between *Sh2d2a* *^−/−^* mice and controls (Figure 4F). In contrast, while the total number of BrdU+ NK cells in spleen remained similar at day 5 post infection *Δm157* MCMV (Figure 4G), a lower proportion of spleen NK cells were BrdU+ in TSAd deficient mice (Figure 4H). This suggested reduced NK cell proliferation in the absence of TSAd in *Δm157* MCMV infection. The reduced BrdU incorporation, in the presence of similar NK cell numbers, indirectly implicates a higher turnover of NK cells in the wild type animals. No difference in cell number but a difference in the proportion of BrdU+ CD8+ T lymphocytes was also observed in *Sh2d2a* *^−/−^* mice during *Δm157* MCMV infection (Figure 4I–K). The decay in NK cells 5 days post-infection was previously described during infection with *Δm157* MCMV^28^,^30^. Here we show that at this early time-point after infection CD8+ T cells display normal levels both in wild-type and *Sh2d2a* *^−/−^* mice (Figure 4J), possibly because CD8+ T cells have already recovered. No significant differences were observed in the numbers or frequency of BrdU uptake in CD4+ T cells (data not shown).

To examine whether the proliferation defect in *Sh2d2a* *^−/−^* NK cells was at the level of receptor repertoire and recognition, the fractions of NK cell populations expressing the activating or inhibitory NK cell receptors KLRG1, Ly49A, Ly49C, Ly49D, Ly49H and NKG2A on the surface were measured by flow cytometry. No significant differences between *Sh2d2a* *^−/−^* mice and their wild type littermates were observed for these receptors (Figure 4L). The decreased BrdU+ incorporation in NK cell and CD8+ T cells from *Sh2d2a* *^−/−^* mice could be the result of an increased cell turnover or an increased migration towards peripheral target organs of MCMV infection. NK cells from the spleen migrate via a chemokine-cytokine-chemokine pathway towards the liver. The liver is an important organ of MCMV replication, and at later time-points during infection, NK cells and CD8+ T cells have been shown to contribute to the control of virus titer in this organ.

### Normal susceptibility to herpes simplex virus 1, coxsackievirus B3 and influenza virus infections in TSAd deficient mice

To further explore the role of TSAd in anti-viral immune responses, three additional viral infection models were explored: herpes simplex virus 1, coxsackievirus B3 and influenza virus. Quite different in their genomes and inducing qualitatively different host responses; all three viruses have a strong public health impact and their clearance is mostly mediated by T cells and NK cells. No differences between *Sh2d2a* *^−/−^* mice and their wild type littermates were observed in the resistance towards these three viruses (Suppl. Figure 1).

### Conclusion

There is currently no evidence that TSAd directly modulates NK cell efficacy in clearing viral infections. Indeed, this study revealed no difference in resistance to MCMV, herpes simplex, coxsackie virus or influenza virus of *Shd2a^−/−^* mice. Interestingly, infection with the *Δm157* MCMV variant yielded higher viral titers in the spleens but not livers of *Shd2a^−/−^* mice. Our results also suggest a reduced proliferative response in spleen NK cells in TSAd deficient mice. Taken together with the observed normal clearance of wild type MCMV, our data indicate that TSAd does modulate NK cell function, but is not necessary for activation of NK cells through the Ly49H activating receptor.

The reason why *Sh2d2a* *^−/−^* mice did not show a defect in viral clearance in the liver upon *Δm157* MCMV infection could be the tissue specific antiviral role of NK cells. For example NK cells from salivary glands, an important reservoir of persistent MCMV infection, are hyporesponsive^34^. Equally, even in MCMV-resistant mice at relatively early times post-infection, which is those that were chosen for this study, control of virus replication by NK cells occurs principally in the spleen^35^.

In response to MCMV infection, NK cells secrete cytokines, display cytotoxic activity and proliferate. In the liver MCMV induces an innate cytokine/chemokine network involving the recruitment of MIP-1α producing macrophages. MIP-1α is a chemoattractant also for INFγ producing NK cells, which are able to control virus load in the liver starting at around day 6^36^,^37^. It is therefore likely that any defect in viral clearance in the liver would be observed only after day 5, which was examined in this study.

Important biological functions can often be difficult to reveal in knock-out models because of functional redundancies. This may also be the case of TSAd, thus other signaling proteins and alternate pathways may have compensated for the lack of TSAd in these experiments. Although more advanced models could help reveal the mechanistic role of TSAd in NK cells in virus infections, our data indicate that TSAd is involved in modulating viral clearance, and provide a direction for further dissecting the observed susceptibility in the context of *Δm157* MCMV infection.

## Methods

### Ethical Statement

Human PBMC were collected with informed consent from healthy blood donors. Collection and analysis of samples were approved by and performed in accordance with the guidelines of the regional ethical committee. The animal protocols and experiments were approved by and performed in accordance with the guidelines of the National Animal Research Authority (Oslo, Norway), the Canadian Council on Animal Care (CCAC) and the McGill University Animal Resources Center.

### Mice

The *Sh2d2a* *^−/−^* C57BL/6 mice have been previously described^2^,^6^. Briefly *Sh2d2a* knockout mice were backcrossed on a C57BL/6–129 background (N8, kindly provided by Professor Jeffrey Bluestone^2^) to C57BL/6 (purchased from the Norwegian Institute of Public Health) for more than 10 generations and then to homozygosity for the disrupted *Sh2d2a* allele.

### In vitro TSAd expression

Mouse NK cell cultures were generated by culturing total splenocytes in recombinant human IL-2 (1000 U/mL) for 6 to 8 days. The proportion of NK cells was then verified by flow cytometry with more than 90% purity (CD49b (DX5)+, CD3-). 2 × 10^6^ cells were left unstimulated, or stimulated for indicated times with PMA (100 ng/ml) and ionomycin (1 μg/ml) then lysed in presence of protease inhibitors, and phosphatase inhibitors before being subjected to western blotting.

Human NKL cells were grown in complete RPMI-1640 media (10% fetal bovine serum, 1 mM sodium pyruvate, 10 mM HEPES, 1% Pen/strep; Wisent) with the addition of 200 U/ml of IL-2. 2 × 10^6^ cells (1 × 10^6^ cells/ml) human PBMC and NKL cells were stimulated with 100 ng/ml of PMA and 500 ng/ml of ionomycin for different time intervals depicted in the figure legends. For western blot analysis, cells were lysed using 0.1% LDS-1% Triton X-100 solution containing 50 mM HEPES, 0.05 M lithium chloride, 0.5 mM PMSF, 2.5 mM EDTA (pH 8.0), 1 mM sodium vanadate and 1% Triton. The cells were lysed with LDS for 15 min and then with Triton for 30 min. After lysis, the cells were sonicated, boiled with SDS loading buffer before proteins were separated by SDS-PAGE and transferred to PVDF membrane (Bio Rad) using a Hoefer Semi-Phor Semi-Dry transfer unit (Amersham Biosciences). The membranes were blocked with Tris-buffered saline (pH 7.4) containing 0.1% Tween 20 (Sigma Aldrich) and 5% skimmed milk, and subsequently incubated with Anti-TSAd (Origene), Anti-GADPH (Chemicon) or Goat anti Mouse (Jackson) in blocking buffer containing 1% skimmed milk. Signals were detected using ECF substrate (GE Healthcare, Pittsburgh, PA, USA) and Storm 860 (GE). Densitometry analysis was performed on the Western blot data using ImageJ.

### MCMV viruses, Infections and Viral Loads

Salivary gland MCMV was prepared by passaging the virus (Smith strain ATCC VR-1399) twice in 3-week old BALB/c mice. The virus was prepared from a homogenate of salivary glands 21 days p.i. Mice aged between 7 and 9 weeks were infected intravenously with 8,000 PFUs of MCMV for 3 or 5 days. The tissue culture-grown viruses^38^ *Δm157* MCMV, which lacks the m157 open reading frame (ORF), have been previously described^39^. Mice were infected with 2 * 10^6^ PFU of *Δm157* MCMV for 5 days. Viral titers of the stock virus or mouse organs (spleen and liver) were evaluated *in vitro* by standard plaque assays on a confluent BALB/c mouse embryonal fibroblast monolayer, as previously described^40^.

### Flow cytometry

For analysis of PBMC cells by flow cytometry after stimulation, the cells were stained for surface markers (CD3-PE (Immunotools) and CD56-PE-Cy7 (eBioscience)) for human PBMC in PBS containing 2% FCS and 0.1% NaN_3_ at 4°C. Cells were then fixed in 2% paraformaldehyde and permeabilized in 0.1% saponin before intracellular staining with anti-TSAd-Dyelight 488 (3C7) from Origene. For analysis of splenic leucocytes from mice, spleens from MCMV-infected and uninfected mice were removed aseptically and gently mashed through a 70 μm nylon mesh (BD Bioscience). Red blood cells were lysed with Ack's Lysis Buffer and the remaining cells were resuspended in the FACS staining buffer. All cells were incubated with 2.4G2 antibodies to block Fc receptors prior to staining, then stained with extracellular Ab, fixed, permeabilized and stained for intracellular antigens using BD cytofix/cytoperm kit according to the manufacturer's protocol. To detect incorporated BrdU on NK and T cells, mice were injected i.p. with BrdU (sigma) 3 hours before sacrificing and collecting the spleen. The cells were first blocked with 2.4G2 antibody and stained for surface antigens and then fixed, permeabilized, treated with DNase I, and stained with FITC-conjugated anti-BrdU antibody (clone 3D4; BD Biosciences), according to the manufacturer's protocol. All the antibodies used in this work were purchased from e-Bioscience, Biolegend or R&D systems, CD3 (2C11), CD4 (GK1.5), CD8 (53–6.7), B220 (RA3-6B2), CD11b (M1/70), CD27 (LG.3A10), DX5 (CD49b), NKp46 (CD335), Ly49A/D (12A8), Ly49C/F/H/I (14B11), Ly49D (4E5), NKG2A/C/E (20d5), KLRG1 (2F1), Ly49H (3D10), IFNg (XMG1.2).

### *In vitro* Cell Stimulations

Splenocytes were extracted and prepared as described above. Prior to 2.4G2 blocking and staining, cells were aliquoted into a 96-well 2HB Immulon plate that had been coated with purified antibody against NK1.1, NKp46, Ly49H, Ly49D, a cocktail of all mentioned antibodies or PMA/Ionomycin or IL12 + IL18 as a positive control. Cells were stimulated in complete RPMI-1640 media with protein transport inhibitors (GolgiStop or Golgiplug; BD). Cells were then incubated for 5 h at 37°C, and then extracellular and intracellular staining was performed as described in the FACS section.

### Herpes Simplex Virus 1 Infection and Phenotyping

Details of the HSV-1 infection procedure have been previously described^41^. Briefly, mice 7 weeks of age or older were, when indicated, infected intraperitoneally (i.p.) or intranasally (i.n.) with 1.10^4^ or 5.10^3^ PFU of HSV-1 strain 17, respectively. In the i.n. inoculation model, mice were anesthetized prior to infection with 5 mg/ml ketamine and 15 mg/ml xylazine. Infected mice were monitored 1–2 times daily over a 2-week period for survival and weight loss. Mice that succumbed to infection within 14 days post-infection (p.i.) were considered susceptible to HSV-1.

### Coxsackievirus B3 Infection and Phenotyping, weight loss, PA and Myocarditis Score

Mice were inoculated intraperitoneally with 10 PFU of CVB3 H3 (generously provided as a plasmid by the laboratory of Dr. Kirk Knowlton^42^) per gram of body weight at 7–8 weeks of age. Animals were weighed daily and at day 8 post-infection, hearts were removed aseptically and sagittally bisected. Heart viral titers were quantified as previously described^43^. Heart sections were fixed in 10% formalin and embedded in paraffin. Samples were sent to the Histology core at the University of Montreal where sections were prepared. Contiguous heart sections were fixed on slides and stained with hematoxylin and eosin (H&E) for evaluation of myocarditis as previously described^44^.

### Influenza Infection and weight loss

Mice were anesthetized with a ketamine/xylazine mixture and infected via intra-nasal inoculation. Each mouse received a weight-adjusted dose of 68 PFU/g of the mouse adapted influenza strain A/HK/1/68-MA20. This strain was derived from a H3N2 clinical isolate of seasonal human influenza from the 1968 Hong Kong pandemic, as previously reported^45^. The bodyweight of animals was monitored daily for two weeks following infection. Mice presenting respiratory distress were humanely sacrificed.

## Author Contributions

P.M. and G.A. designed, performed experiments, analyzed data and wrote the main manuscript text. N.F. designed and performed experiments and analyzed data, R.G., P.S., S.W., G.B. and G.C. performed experiments. M.M. analyzed data, E.D., A.S. and S.V. designed experiments, analyzed data and contributed to the writing of the paper. All authors reviewed and approved the final version of the manuscript.

## Supplementary Material

Supplementary Information Supplementary figure 1
