# Supplementary material for: Reduced MCMV Δm157 viral clearance in the absence of TSAd
Source: Sci Rep. 2015 Mar 18;5:9219. doi: 10.1038/srep09219 (PMC4363830; doi:10.1038/srep09219)
Supplement: Supplementary Information — Supplementary figure 1 [file srep09219-s1.pdf]

Reduced MCMV Δm157 viral clearance in the absence of TSAd.

Moussa P* [1], Abrahamsen G* [2], Fodil N [1], Gopalakrishnan RP [2], Mancini, M [1], Dissen E [2], Sæther PC [2], Wiltshire SA [1], Boivin GA [1], Caignard G [1], Spurkland A [2**], Vidal SM [1]

[1] Department of Human Genetics and Department of Microbiology and Immunology, McGill University, Life Sciences Complex Montreal, QC, Canada

[2] Department of Anatomy, Institute of Basic Medical Sciences, University of Oslo, Oslo, Norway.

The authors declare no conflict of interest.

* These authors contributed equally to this work

**Corresponding author
Anne Spurkland
Institute of Basal Medical Sciences
University of Oslo
P.O. 1105 Blindern
0317 Oslo
Norway
E-mail: anne.spurkland@medisin.uio.no,
Phone: +47 22851125, Fax: +47 22851278

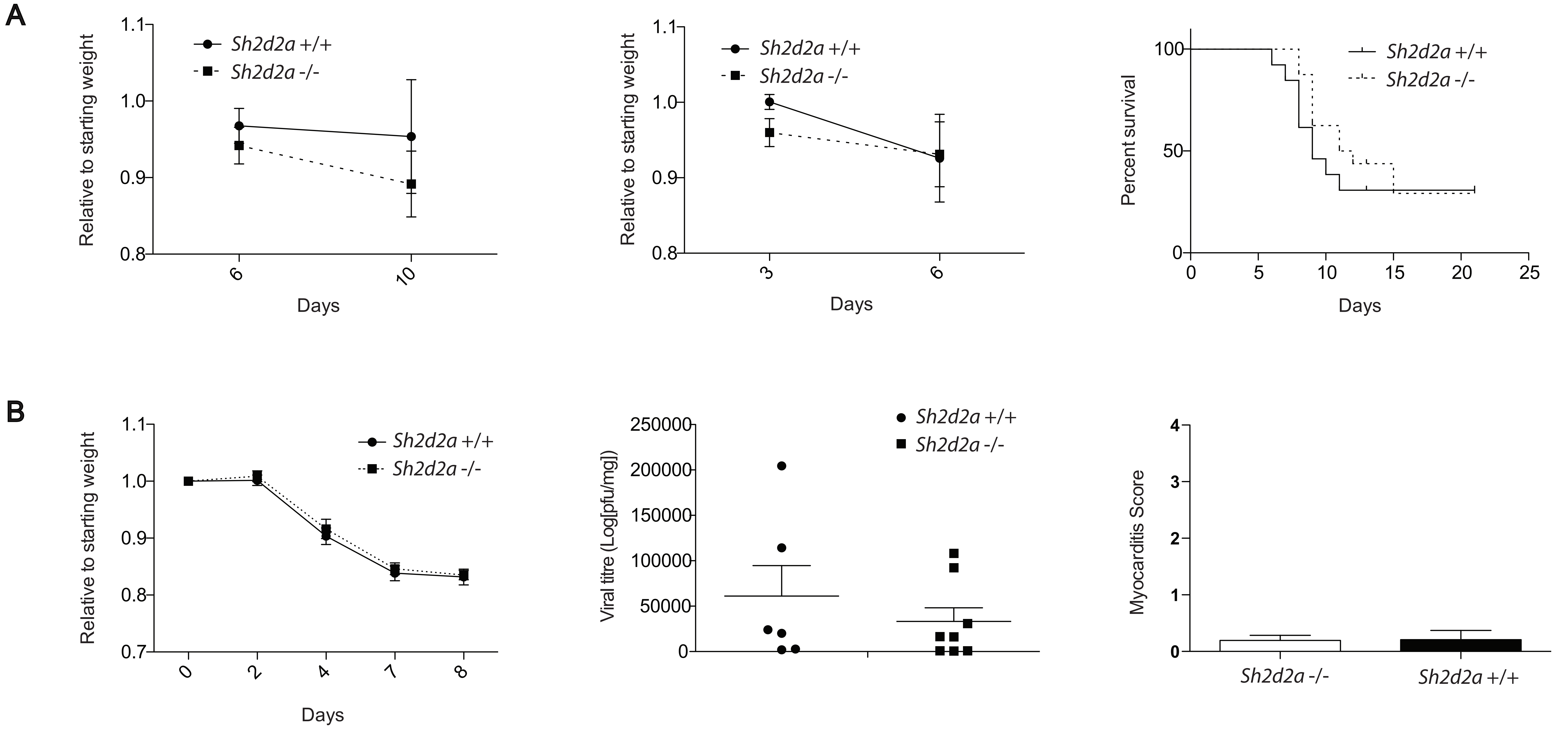

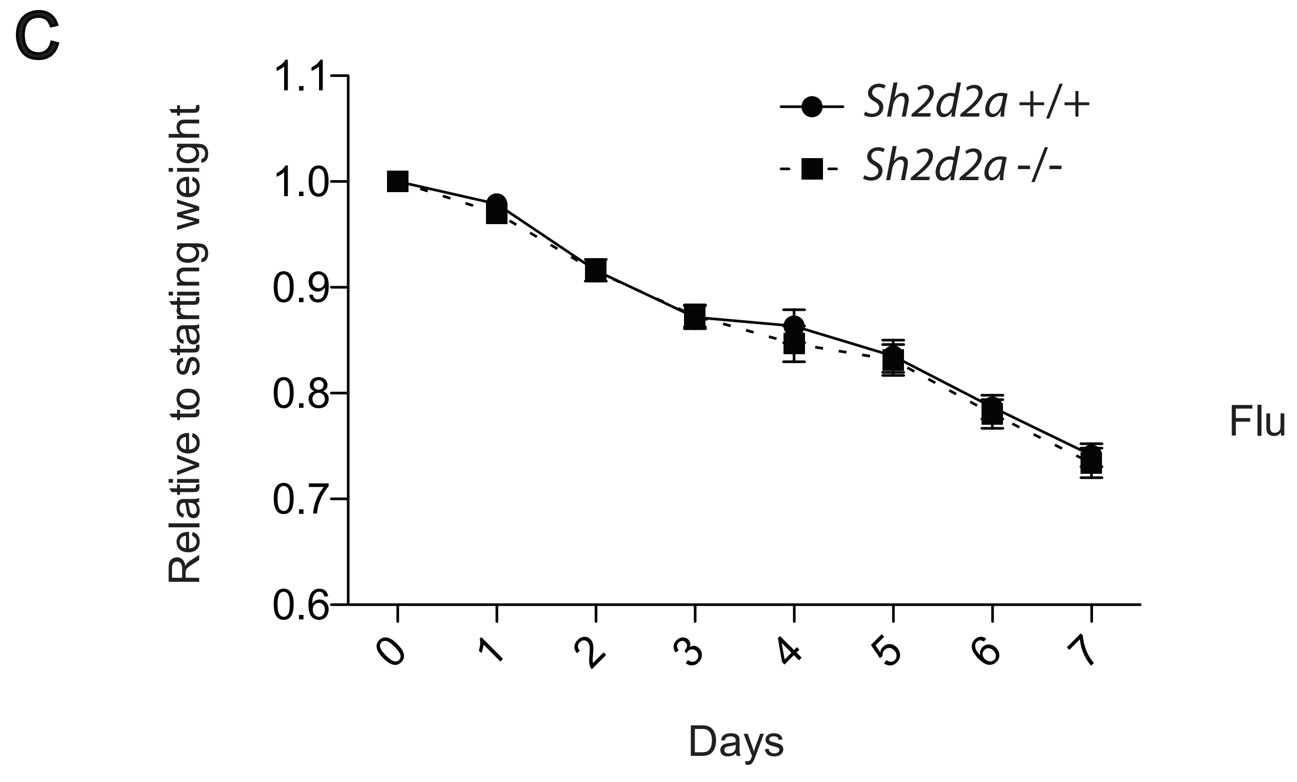

Supplementary figure 1: Various viral infections in *Sh2d2a* +/+ and *Sh2d2a* -/- mice and their phenotypic outcome. a) HSV1 infections. The leftmost panel shows weight loss due to I.P infections using 10^4 PFU. The middle panel shows weight loss due to HSV1 I.N infection using $5*10^4$ PFU. The rightmost panel shows survival post I.P infections using 10^4 PFU. b) CVB3 infections. The leftmost panel shows weightloss due to IP infection with 10PFU/g of mouse. The middle panel are viral titers day 8 post infection with 10PFU/g of mouse and the rightmost panel are myocarditis scores under identical infection conditions. c) Weight loss due to influenza infection with 1500PFU/22g of mouse .
